# Peer support for family carers of people with dementia, alone or in combination with group reminiscence in a factorial design: study protocol for a randomised controlled trial

**DOI:** 10.1186/1745-6215-12-205

**Published:** 2011-09-15

**Authors:** Georgina Charlesworth, Karen Burnell, Jennifer Beecham, Zoë Hoare, Juanita Hoe, Jennifer Wenborn, Martin Knapp, Ian Russell, Bob Woods, Martin Orrell

**Affiliations:** 1 Research Department of Clinical, Educational, and Health Psychology, University College London, 1-19 Torrington Place, London, WC1E 7HB, UK; 2 Research and Development Department, North East London NHS Foundation Trust, Goodmayes Hospital, Barley Lane, Ilford, Essex, IG3 8XJ, UK; 3 Mental Health Sciences Unit, University College London, Charles Bell House, 67-73 Riding House Street, London, W1W 7EJ, UK; 4 Personal Social Services Research Unit, London School of Economics and Political Science, Houghton Street, London, WC2A 2AE, UK; 5 North Wales Organisation for Randomised Trials in Health, Institute of Medical & Social Care Research (IMSCaR), Holyhead Road, Bangor University Bangor, Gwynedd, LL57 2PZ, UK; 6 Health Services and Population Research Department, Institute of Psychiatry, King's College London, UK; 7 West Wales Organisation for Rigorous Trials in Health, Swansea University, College of Medicine, Institute of Life Sciences, Singleton Park, Swansea, SA2 8PP, UK

## Abstract

**Background:**

Peer support interventions can improve carer wellbeing and interventions that engage both the carer and person with dementia can have significant mutual benefits. Existing research has been criticised for inadequate rigour of design or reporting. This paper describes the protocol for a complex trial that evaluates one-to-one peer support and a group reminiscence programme, both separately and together, in a factorial design.

**Design:**

A 2 × 2 factorial multi-site randomised controlled trial of individual peer support and group reminiscence interventions for family carers and people with dementia in community settings in England, addressing both effectiveness and cost-effectiveness.

**Discussion:**

The methods described in this protocol have implications for research into psychosocial interventions, particularly complex interventions seeking to test both individual and group approaches.

**Trial Registration:**

ISRCTN37956201

## Background

People with dementia who are cared for by a family member are less likely to be hospitalised or move into residential care [1], and have a better quality of life than those in care homes [2]. However, family carers of people with dementia experience greater strain and distress than carers of other older people [3]. Family carers may experience social isolation [4] through lack of personal time and opportunities to socialise, and stigma resulting in family and friends distancing themselves [5]. Without support, family carers can feel emotionally and physically burdened and may experience interrelational conflicts, which may reduce perceived level of emotional support and increase feelings of loneliness [6]. As interventions delivered by health staff can 'medicalise' the experience of caring and raise stigma [7], interventions in the voluntary and community sector can provide a more informal setting for the support of carers. The World Health Organisation noted the importance of enhancing social relationships for carers [8], and peer support for carers is included in the recommendations of the National Dementia Strategy for England [9]. It has been suggested that peer support can directly improve wellbeing by decreasing feelings of isolation and/or encouraging more appropriate coping strategies, and enabling a change in behaviour, emotion or cognitions [10]. Hence a body of work is developing to evaluate befriending and peer support interventions.

### Befriending and peer support interventions

A recent systematic review of befriending interventions in healthcare established that befriending has a modest effect on depressive symptoms. Interventions tend to be short-term (around 3 months) and are often delivered by professionals sometimes in collaboration with lay volunteers [11]. For family carers of people with dementia neither the effectiveness nor the cost-effectiveness of befriending or peer support has been established. A large Randomised Controlled Trial (RCT) of befriending found modest effects of long-term befriending, but was not cost-effective [12,13].

However, carers reported that they enjoyed and gained from the experience, particularly those who were befriended by former family carers, which highlights the potential therapeutic effects of peer support. Indeed, research has found that peer support can help carers in particularly stressful caring situations [14].

### Relationship focused

In contrast to carer-focused interventions there is growing evidence that carer wellbeing may be enhanced through interventions that engage both the primary carer and the person with dementia [15,16], often described as dyadic interventions. Such interventions include the 'Remembering Yesterday Caring Today' reminiscence group programme [RYCT; [17]] which has been widely used across Europe and is being evaluated in a large UK RCT [18].

## Aim

The present trial is one of three psychosocial interventions being carried out as part of the Support at Home: Interventions to Enhance Life in Dementia (SHIELD) research programme (NIHR grant RP-PG-060-1083). This trial evaluates the effectiveness and cost-effectiveness of the SHIELD Carer Supporter Programme (CSP) and RYCT, both separately and combined. The trial is described here in accordance with the revised CONSORT 2010 reporting requirements [19], as applied to pragmatic trials [20]. Developing trial designs for complex interventions is challenging, and the MRC guidance on complex interventions [21] emphasises the importance of selecting or developing a research design that can evaluate interventions appropriately and efficiently. The challenge for this trial was to find a scientifically valid, but logistically feasible, method for comparing contrasting interventions where one had an individual focus, and the other a relationship focus within a group setting.

## Design

**Figure 1:**
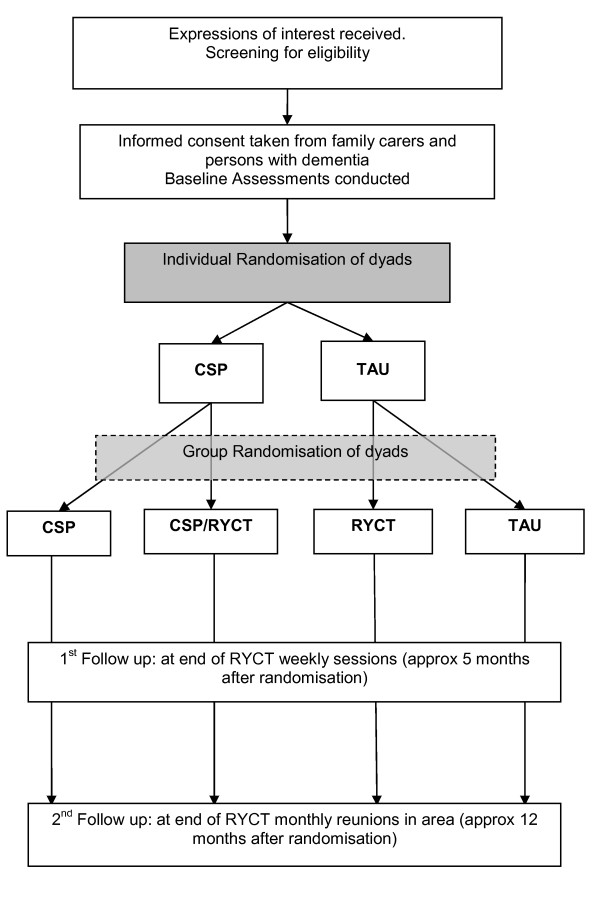
**Flow diagram of the SHIELD CSP-RYCT trial**. A two-stage sequential dynamic algorithm is used in which participants are first randomised individually to CSP or TAU and later on a group basis to RYCT or TAU.

The trial is a 2 × 2 factorial single-blind RCT (Figure 1): the 4 arms of the trial are CSP alone, RYCT alone, CSP and RYCT combined, and treatment as usual (TAU). To ensure enough participants to run viable RYCT groups, we randomise between TAU, RYCT, CSP and combined in the proportions 1:2:1:2. We collect data at baseline (post-consent, pre-randomisation), and 5 and 12 months after the first randomisation, with the main endpoint at 12 months. After randomisation, all participants are free to continue to receive treatment as usual from statutory and voluntary services in their locality. We conducted a feasibility study before the full RCT in accordance with MRC guidance on complex interventions guidance [21].

The design tests three null hypotheses:

i) There is no effect of CSP compared with TAU

ii) There is no effect of RYCT compared with TAU

iii) There is no interaction between RYCT and CSP

### Setting

We are running the trial in community settings in North East London, Norfolk, Northamptonshire, and Berkshire.

### Participants

#### Eligibility criteria

All participants are adult (18 years and over) English-speaking carers for a relative or close friend living at home in the community with a primary progressive cognitive impairment or dementia as defined by DSM-IV criteria for dementia [22]. Carers have to agree to take part in the full trial (with the right to withdraw from the research at any time) and for the person they care for to be approached to take part. Carers are excluded if they or the person they care for have a congenital learning disability or non-progressive brain injury. Carers with a diagnosed terminal illness are also excluded as are those already taking part in another psychosocial intervention study.

#### Recruitment strategy

Recruitment is both direct and indirect to address inherent difficulties in recruiting carers and people with dementia [23]. Direct recruitment targets participants within the community using leaflets, flyers and posters. Strategies for newer and 'hard-to-reach' carers include invitations in local papers and newsletters. Indirect recruitment uses gatekeepers, like the Alzheimer's Society and Admiral Nurses already in contact with the target population. These gatekeepers tell potential participants about the study and distribute recruitment literature. Contact details of potential participants are only passed to the research team where consent has been given for this.

### Involving Service User and Carers

We developed the CSP intervention and supporting related documentation in consultation with service users and carers [24]. Former and experienced family carers also have involvement as direct providers of the CSP element of the trial.

### Interventions

Each intervention is organised and provided independently of the research assessments.

#### Peer Support (SHIELD Carer Supporter Programme)

SHIELD CSP gives newer carers access to an adult Carer Supporter (CS), who is an experienced family carer or close friend of a person with dementia. The intervention was originally based on a model of collaboration and partnership with a local voluntary carer support organisation [25]. The research team worked with a Carer Supporter Manager (CS Manager), based within a host voluntary sector organisation, to develop the CSP intervention. There is one Carer Supporter Coordinator (CS Coordinator) per trial centre who recruits and screens the CS and is supported by the CS Manager. In two of the four sites the CSP intervention is provided by the host NHS organisation, reflecting 'usual practice' within their area.

Before being matched with a family carer participant, CS volunteers attend a mandatory 'Being a Carer Supporter' orientation and awareness course led by the CSP Programme Lead, the CS Manager and/or relevant CS Coordinator and local health and social care personnel. The programme has six modules: introducing CSP; experiences of dementia and caring; the Carer Supporter role: what it is and what it is not; listening and helping skills; working in other peoples' homes; and dementia awareness for carers and local resources. The CSs must also agree to abide by the Code of Conduct and Statement of Confidentiality.

CSs and family carers are paired through a process of matching based on demographic factors and personal preferences, for example the type of dementia they have experience of, and geographical proximity to one another. We match pairs to encourage longer and more satisfying peer support relationships [26]. The role of the CS is to provide emotional and informational support to the family carer, offer family carers a listening ear, and signpost them to local services and other useful contacts and resources. We ask CSs to support their family carers for at least one hour per week for the first three months, and then reduce the frequency to approximately twice a month for a further seven months. Contact is face-to-face or over the telephone. CSs must not carry out tasks that would otherwise be carried out by a paid worker like nurses or home care workers, or to give advice or respite care. CSs are supported by a CS Coordinator throughout the duration of the match.

#### Group Reminiscence (Remembering Yesterday, Caring Today)

The group reminiscence intervention follows the RYCT programme for people with dementia and their family carers [17]. Twelve weekly sessions, each lasting two hours, cover a range of themes including: childhood and family life; school days; courting and marriage; food and cooking; and the next generation. Each session explores its theme using multisensory triggers and activities, including group discussions, small group activity, object handling, enacting or improvisation, and singing songs. The RYCT intervention is provided by either the NHS host organisation or the host voluntary sector organisation depending on usual practice in each locality. The SHIELD programme runs RYCT in community settings such as church halls, with transport provided if needed. Each group session is led by two experienced Facilitators, supported by a team, including volunteers, health and social care staff and trainees to facilitate small group discussion and activities and engage the people with dementia. All members of the RYCT team must attend a training day led by one of the original RYCT programme authors.

During four of the sessions, the family carers meet together with one of the Facilitators and another team member for about 45 minutes, whilst the other Facilitator and remaining team work with the people who have dementia. The Carers' sessions aim to develop listening and communication skills and consider how the activities and strategies used within the sessions can be carried over into the home environment. After the 12 initial sessions, monthly reunion sessions take place over a further seven months using previously successful themes, or using new themes, depending on the preferences of the group.

#### Combined Intervention (SHIELD CSP/RYCT)

The combined intervention offers both contact with a CS and the opportunity to attend the RYCT programme. CSs matched with family carers who have been randomised to this intervention group are invited to attend the RYCT sessions, RYCT training with other volunteers, and a 2-hour training session. The latter focuses on the purpose of reminiscence and techniques to encourage reminiscence at home, to enable the CS to better support the family carer in implementing the strategies and advice provided within the RYCT Carers' sessions. The aim of this intervention is to extend the benefits of RYCT through the CS bringing knowledge of the care dyad to the group, and then encouraging reminiscence in the family carer's home.

#### Treatment as usual

The aim of this trial is to examine effectiveness and cost-effectiveness of interventions over and above usual care, which varies between sites. Hence participants randomised to the control group continue with any health, social or voluntary sector services they are currently receiving or commence once recruited into the trial. Like the intervention groups, this group is also given a list of useful resources in their area.

#### Implementation of Interventions

The MRC Guidance for Developing and Evaluating Complex Interventions [21] recommends that researchers monitor the extent to which interventions have been delivered to and received by the participants [27]. We have designed measures specifically for this trial that capture:

• Treatment Delivery: the extent to which the intervention provider adheres to treatment protocol, the absence of any other intervention, and the quality of the intervention

• Treatment Receipt: the extent to which the participant receives the intervention, the intensity of the intervention received, and satisfaction with that intervention.

These data are also entered into a MACRO™ database.

### Measures

The primary outcome is health-related quality of life for the carer. Secondary outcomes for the carer include psychological well-being, social support, coping and self-efficacy. Outcomes for the person with dementia include quality of life, well-being, relationship quality, neuropsychiatric profile, activities of daily living, and cognition. To explore the active ingredients or mechanisms of change of the intervention(s) (i.e. how the intervention might work) [21], we ask family carers about the process and experience of caring. To explore cost-effectiveness analysis, we also collect information from them on resource use and carer time inputs.

#### Primary outcome

The primary outcome is the family carers' health-related quality of life, measured by the validated and widely used Mental Health Component Summary (MCS-12) of the UK Short Form-12 Health Survey [UK SF-12; [28]]. The SF-12 measures general health status from the perspective of the participant, and in addition to the MCS-12 also allows for the generation of a second sub-score, the Physical Component Summary (PCS-12). Reliability is 0.74 for MCS-12 and 0.78 for PCS-12 [29]. Validity is 0.97 for MCS-12 and 0.67 for PCS-12 [30].

#### Secondary outcomes

Family carer:

• Health-related quality of life: EQ-5D [31], comprising 5 items and a Visual Analogue Scale (VAS).

• Anxiety and depression: Hospital Anxiety and Depression Scale [HADS; [32]] comprising 14 items - 7 for anxiety and 7 for depression.

• Positive and Negative Affect: Positive and Negative Affect Schedule [PANAS; [33]] comprising two 10-item scales one measuring positive mood, the other negative mood.

• Aspects of caring: Carers of Older People in Europe Index [COPE-Index; [34]] comprising 17 items measuring carers' perceptions of their role, positive and negative aspects of caregiving, and issues of support.

• Emotional loneliness: the 2-item Loneliness Scale [35].

• Relationship quality: Quality of Caregiver-Patient Relationship [QCPR; [36]] comprising 14 items measuring expressed emotion along two dimensions: level of criticism and level of warmth.

• Coping: Brief-Cope [37] comprising 28 items measuring approaches to coping.

• Self-efficacy: Revised Scale for Caregiving Self Efficacy [RSSE; [38]] comprising 15 items to measure self-efficacy across three domains.

• Carer distress: Neuropsychiatric Inventory with Caregiver Distress Scale [NPI-D; [39]] assessing ten behavioural disturbances associated with dementia.

• Social support: The Positive and Negative Social Exchanges [PANSE; [40]] comprising 24 items measuring the frequency of four domains of positive and negative social exchanges.

• Social network: Practitioner Assessment of Network Type [PANT; [41]] comprising 8 items that determine type of supportive network available to the participant.

• Personal growth: The 3-item version Personal Growth Index [PGI; [42]].

Person with dementia:

• Health-related quality of life: EQ-5D [31] - as for family carers, but completed by family carer if person with dementia is unable; this proxy measure does not include the VAS.

• Anxiety and depression: HADS [32].

• Relationship quality: QCPR [36].

• Quality of life in dementia: Two measures are administered:

◦ Quality of Life - Alzheimer's Disease Scale [QoL-AD; [43]] comprising 13 items namely physical health, energy, mood, living situation, memory, family, marriage, friends, chores, fun, money, self, and life as a whole (also completed by the carer).

◦ DEMQOL [44] comprising 29 items covering health, wellbeing, cognitive functioning, social relationships, and self-concept; the carer also completes the 31-item proxy version.

• Cognition: Mini-Mental State Examination [MMSE; [45]] is a widely used rating of cognitive function. Completed by the person with dementia.

• Activities of daily living: Alzheimer's Disease Co-operative Study - Activities of Daily Living Inventory [ADCS-ADL; [46]] assesses functional capacity across a number of daily tasks. Completed by the family carer.

• Global functioning: Clinical Dementia Rating [CDR; [47]] denoting presence and severity of dementia. Completed by the research interviewer(s).

#### Resource Use

The Client Service Receipt Inventory (CSRI) [48] is used to collect information retrospectively about use of health, social care and other relevant services, accommodation and living situation, benefits, carer support, and employment. It provides data from which we calculate costs.

### Sample size

We based sample size calculations on the BECCA [12] and REMCARE [18] trials. These predicted effect sizes, defined as average effect per participant divided by population standard deviation, of 0.42 for CSP and 0.35 for RYCT. In a 2 × 2 factorial design using a 2:1 allocation ratio in favour of groups receiving RYCT, a completed sample of 240 dyads would yield power of more than 90% to detect both main effects using a significance level of 5%. This design would also yield power of more than 80% to detect interaction between CSP and RYCT equivalent to an effect size of 0.4, using an analogous definition. As both the REMCARE trial platform and BECCA retained some 80% of participants, we aim to recruit 300 dyads in 13 rounds of 24 dyads to yield a final sample of 240 dyads.

### Randomisation

We use a two-stage sequential dynamic algorithm to allocate individual and group based interventions. We base the probability of choosing each group on the participants' stratification variables and the previous allocations for those variables. For the first allocation between CSP and TAU the stratification variables are locality and dyad relationship. In the latter, dyads are designated 'horizontal' or 'vertical' kinship, where horizontal denotes spouses and siblings and vertical denotes daughters, sons, daughters-in-law and other similar kinships where there carer and person with dementia are from different generations within the family. For the second allocation between RYCT and TAU we add the first allocation as a stratification variable to keep the four treatment arms in balance.

The SHIELD administrator enters participants' variables into a remote web-based randomisation, which then allocates them in equal proportions between CSP or TAU on an individual basis. Once adequate dyads have been randomised within each site (target 24; range 16-30), the remote randomisation system allocates them in a second randomisation between RYCT and TAU in the proportion 2:1 on a group basis. Hence the combination of these two randomisation stages results in results in the allocation between TAU, RYCT, CSP, and RYCT plus CSP in the proportions 1:2:1:2. The (unblinded) SHIELD administrator then informs carers of their allocation by letter, and liaises with the RYCT Facilitator and/or CS Coordinator as appropriate.

### Blinding

It is not possible to blind participants or providers to their allocated intervention in psychosocial interventions. In this trial, for example, both the CS Coordinator and RYCT Facilitator need to know which carers are allocated to the combined interventions. However, we do blind research interviewers who assess outcomes, in particular by limiting access to the web based management system. Research interviewers recorded their perception of participants' allocations for use as a covariate in statistical analysis.

### Research Governance

Three formal groups meet regularly to manage the trial within the SHIELD programme: the Project Management Group (PMG); the Programme Steering Committee (PSC); and the Data Monitoring and Ethics Committee (DMEC).

### Ethical arrangements

Ethical approval was given by the Outer North East London Research Ethics Committee (09/H0701/54). In line with Good Clinical Practice, we developed a Standard Operating Procedure for accurate and timely reporting of Serious Adverse Events to the Chief Investigator. He assesses whether either intervention could have caused each event and, if so, reports to the DMEC as appropriate.

#### Consent

The main participants are family carers and it is hoped that their relatives with dementia will also take part. They each enter the trial only after giving informed consent. We explain the nature of the research to all participants and give them at least 24 hours to consider the study information before seeking consent, stressing that their care will not change if they choose not to participate. We also tell the general practitioners (GPs) of all participants about their participation. If a person with dementia is considered to lack capacity, research staff, acting in accordance with the Mental Capacity Act 2005 [49] and the British Psychological Society Code of Ethics and Conduct [50], seek documented assent from the family carer. CSs also give informed consent as an acknowledgement of the CSs' Code of Conduct and also because they are participants in a linked project evaluating the impact of volunteering on the volunteer.

### Data management and statistical analysis

Data are entered into Infermed's MACRO™ Electronic Data Capture system for clinical trials that allows data to be both entered and reviewed. Data are managed within MACRO™ and exported to a statistical software package for analysis. Multiple imputation with a linear regression model will be used for imputing missing outcome data.

#### Effectiveness analysis

After exporting data to the statistical package SPSS, we shall follow the general approach of the UK BEAM trial [51] and analyse relevant outcomes on the basis of treatment allocated to test whether:

A. CSP, alone or in combination with RYCT, affected family carers and their relatives;

B. RYCT, alone or in combination with CSP, affected family carers and their relatives;

C. CSP and RYCT interacted in their effects on these outcomes.

Specifically we shall use multi-level analysis of covariance with outcome at 12 months after initial randomisation as dependent variable and 'outcome' at baseline and length of time on CSP as covariates, to compare: (A) CSP and combined groups with TAU and RYCT groups; (B) RYCT and combined groups with TAU and CSP groups; and (C) combined and TAU groups with CSP and RYCT groups. We shall also use linear mixed models to study longitudinal effects at 5 and 12 months. We shall treat RYCT versus TAU and CSP versus TAU as fixed effects, together with gender and dyad relationship. We shall treat the effects of locality (including personnel) and time as random effects and assess the covariance structure of this. We shall undertake sensitivity analysis to test whether plausible changes in key variables such as carers' ages and relationships to their relatives with dementia would have affected findings.

#### Cost-effectiveness analysis

The primary health economic evaluation will estimate the incremental cost-effectiveness of:

A. CSP compared with TAU, both alone or both in combination with RYCT;

B. RYCT compared with TAU, both alone or both in combination with CSP;

C. CSP and RYCT combined compared with TAU.

These analyses will take a societal perspective, and measure effectiveness on the MCS-12 [28]. In principle each cost-effectiveness analysis will have five stages: (a) measurement of outcomes; (b) measurement of costs; (c) estimation of incremental cost-effectiveness ratio (ICER); (d) calculation of net benefits and derivation of cost-effectiveness acceptability curve (CEAC); (e) and sensitivity analyses. As these three analyses have many possible combinations of outcomes we shall follow the standard economic evaluation approach of extended dominance.

#### Outcome estimation

The economic evaluation will employ the same procedures for calculating scores and imputing missing values as in the primary statistical analyses described above.

#### Cost estimation

The economic evaluation will use responses on the validated CSRI [48] completed with the carer. We will attach nationally relevant unit costs to services used by participants, using the most recent Personal Social Services Research Unit 'Unit Costs' volume where available [52], and costed locally where not, for example for CSP and RYCT. The time inputs of carers and their lost productivity (from giving up or reducing employment) will be costed using a range of methods to reflect the opportunity costs to those individuals, and the consequences of differences explored in sensitivity analyses. The primary economic analysis will include costs, both of service use and of carer inputs (a societal perspective).

We shall compare the outcomes and costs of the four allocated groups. For example, we shall interpret CSP as more cost-effective than TAU (both alone or both in combination with RYCT) if: (i) it is both less costly and more effective on the MCS-12; or (ii) it is both more costly and more effective, and the decision maker values the improvement in outcome at more than the additional cost; or (iii) it is both less costly and less effective and the decision-maker attaches lower value to the loss of outcome than the cost savings. In the fourth option, when CSP is more costly and less effective than TAU, the decision maker-would prefer TAU.

To quantify options (ii) and (iii) in this example, we shall estimate the ICER, namely the mean cost difference between CSP and TAU (both alone or both in combination with RYCT) over the follow-up period of 12 months divided by the mean difference between these groups in the MCS-12 over those 12 months. We will also estimate net benefit (NB) for each dyad from the standard formula:

NB=λ×E-C

where:

E is effectiveness, namely the change in the primary outcome; C is the cost for that dyad (services used by the carer and the person with dementia, plus indirect costs of carer time); and λ is the decision-maker's willingness to pay for one additional unit of outcome. After calculating net benefits for each dyad for plausible values of λ, we shall address the expected skewness in the distribution of NBs by 'bootstrapping'; this is the technique of drawing (say) 10,000 independent replicates with replacement from the original sample of (say) 240 dyads while keeping the four allocated groups at their original sizes. For each value of λ we shall use these replicated samples to estimate the true proportion of dyads whose net benefit is positive, and derive a CEAC plotting the likelihood of CSP being cost-effective relative to TAU (both alone or both in combination with RYCT) against λ, the decision-maker's willingness to pay. We shall conduct sensitivity analyses to explore the effect on conclusions of varying key assumptions about costs and outcomes.

#### Secondary cost-effectiveness analyses

A number of secondary economic analyses will be conducted.

The cost-effectiveness analyses set out above (primary analyses) will be repeated from a health and social care system perspective, which in this case will mean looking only at costs to the health and social care system, but still focusing on MCS-12 as the outcome measure.

We will use the EQ-5D for carers to calculate utility using societal preference weights, and examine cost-effectiveness with outcomes measured as utility (quality-adjusted life years), first from a societal perspective and then from a health and social care system perspective. The stages of analysis will match exactly the approach to be adopted for the primary cost-effectiveness analyses described above. The results from these analyses will be in the form of cost per additional quality-adjusted life year (QALY) and will permit comparison with studies in other clinical fields and with (for example) NICE recommendations.

Other outcomes (for carers and people with dementia) will be examined alongside costs in a series of cost-consequences analyses to cast further light on the impact of the interventions.

## Discussion

The design of the trial is unusual since there are few psychosocial intervention studies that merit a factorial design; compare individual, group, and combined interventions; and undertake a cost-effectiveness analyses, particularly where the unit of consideration is the dyad rather than the carer. We know of no examples of factorial studies that assess the impact of interventions on the mental health of family carers. Hence this pragmatic trial has the potential to inform and potentially influence policy and practice not only in relation to care and treatment for dementia, but also in other chronic mental and even physical illnesses. The SHIELD CSP-RYCT trial aims to evaluate peer support focused on both the carer through CSP and dyad through RYCT. This combination seeks to support the family carers, their relatives with dementia, and the relationship they share. Supported carers have the potential to care for the person with dementia to provide them with a better quality of life than those in residential care [2] coupled with economic advantages.

## Competing interests

The authors declare that they have no competing interests.

## Authors' contributions

Contributions: GC, MO, and BW developed the original concept for the trial; GC and KB drafted the original protocol; GC and IR developed the design and methodology; MK developed the health economic component; GC developed the CSP; GC, BW and JW developed the combined intervention; BW led the development of the original RYCT publication; JW wrote the RYCT implementation manual for CSP-RYCT; IR advised on the trial design and methods and ZH developed the analysis plan; KB and GC adapted the trial proposal as a protocol paper; all authors reviewed and commented on drafts of the protocol paper.
